# Tumor suppressor berberine binds VASP to inhibit cell migration in basal-like breast cancer

**DOI:** 10.18632/oncotarget.9968

**Published:** 2016-06-13

**Authors:** Ke Su, Pengchao Hu, Xiaolan Wang, Changchun Kuang, Qingmin Xiang, Fang Yang, Jin Xiang, Shan Zhu, Lei Wei, Jingwei Zhang

**Affiliations:** ^1^ Department of Nephrology, Renmin Hospital of Wuhan University, Wuhan 430060, P. R. China; ^2^ Department of Pathophysiology, School of Medicine, Wuhan University, Wuhan 430071, P. R. China; ^3^ Department of Pathology, Shanghai First People's Hospital, Shanghai 200080, P. R. China; ^4^ Department of Pharmacy, Wuhan General Hospital of Guangzhou Command, Wuhan 430070, P. R. China; ^5^ Department of Plant Science, College of Life Sciences, Wuhan University, Wuhan 430072, P. R. China; ^6^ Department of Pharmacology, College of Pharmacy, Wuhan University, Wuhan 430071, P. R. China; ^7^ Department of Breast and Thyroid Surgery, Renmin Hospital of Wuhan University, Wuhan 430060, P. R. China; ^8^ Department of Oncology, Zhongnan Hospital of Wuhan University, Wuhan 430071, P. R. China

**Keywords:** berberine, basal-like subtype breast cancer, VASP, polymerization

## Abstract

Berberine is a plant-derived compound used in traditional Chinese medicine, which has been shown to inhibit cell proliferation and migration in breast cancer. On the other hand, vasodilator-stimulated phosphoprotein (VASP) promotes actin filament elongation and cell migration. We previously showed that VASP is overexpressed in high-motility breast cancer cells. Here we investigated whether the anti-tumorigenic effects of berberine are mediated by binding VASP in basal-like breast cancer. Our results show that berberine suppresses proliferation and migration of MDA-MB-231 cells as well as tumor growth in MDA-MB-231 nude mouse xenografts. We also show that berberine binds to VASP, inducing changes in its secondary structure and inhibits actin polymerization. Our study reveals the mechanism underlying berberine's inhibition of cell proliferation and migration in basal-like breast cancer, highlighting the use of berberine as a potential adjuvant therapeutic agent.

## INTRODUCTION

Breast cancer is the most common malignancy in women worldwide and its mortality rate is second to cervical cancer in developing countries [1]. Over the past 30 years, the incidence and mortality of breast cancer have been increasing, with a rate that is second only to lung cancer [1]. The incidence of breast cancer in China is relatively low compared to that in other countries, but soared from 30,000 cases per year in the late 1980s to 67,000 in the early 1990s [2].

Gene *expression profiling* has allowed to classify *breast cancer into four subtypes* [3]: luminal A (ER- and/or PR-positive, HER-2-negative, Ki-67 expression less than 14%), luminal B (ER- and/or PR-positive, HER-2-negative, Ki-67 expression more than or equal to 14%; or ER- and/or PR-positive, HER-2 overexpression, Ki-67 at any level), basal-like (ER- and PR-negative, HER-2-negative), and HER-2 overexpression (ER- and PR-negative, HER-2 overexpression). Luminal A is the most common type of cancer (~46% of cases), followed by basal-like (~28.8%), luminal B (~14.7%) and HER-2 overexpression (~10.4%)[4, 5]. These four molecular subtypes of breast cancer differ in phenotype, response to drugs, and survival rates, thus requiring different treatments. Basal-like breast cancer is characterized by larger, high-grade tumors, with higher risk of lymph node and distant metastasis than the other subtypes [4, 6–8]. While the luminal and HER-2 overexpression types of breast cancer are responsive to targeted treatments, systemic chemotherapy is still the main form of routine treatment for basal-like breast cancer [9], which is characterized by poor prognosis [10].

The alkaloid berberine (berberine, BBR) can be extracted from multiple plants and has been long used as a non-prescription drug to treat diarrhea. In addition, pharmacological and clinical studies have demonstrated other beneficial effects from berberine treatment [11], including inhibition of tumor growth in a variety of cancer types [12–15]. In addition, berberine can inhibit invasion and metastasis of breast cancer MCF-7 (luminal A type) and MDA-MB-231 cells (basal-like type) [16–18].

Previously, our group showed that the actin-associated protein vasodilator-stimulated phosphoprotein (VASP) promotes cell proliferation, adhesion, motility and cell migration [19, 20], and is overexpressed in high-motility breast cancer cells, that is to say, VASP expression was higher in MDA-MB-231 cell than that in MCF-7 cell [17]. Furthermore, knocking down VASP expression in breast cancer cells inhibits cell migration and invasion [17]. Thus, inhibition of cytoskeletal rearrangements by targeting VASP-induced actin assembly could have therapeutic benefits in basal-like breast cancer.

In this study, we evaluated the effects of berberine treatment on MDA-MB-231 and MCF-7 breast cancer cells *in vitro* and *in vivo*. We found that berberine binds to VASP and inhibits actin filament elongation, especially in basal-like breast cancer cells.

## RESULTS

### Expression of VASP in different breast cancer subtypes

**Figure 1:**
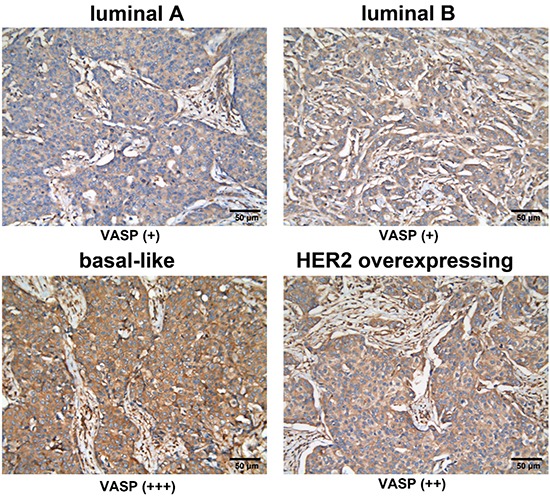
VASP expression in four subtypes of breast cancer Immunohistochemical analysis of VASP in different subtypes of breast cancer. luminal A: weakly positive (+); luminal B: weakly positive (+); basal-like: strongly positive (+++); HER-2 overexpression: moderately positive (++). (200× magnification).

First, we performed immunohistochemical analysis of VASP in 41 breast cancer samples. Among the different subtypes, strong expression (+++) of VASP was present in 25.0% (2/8) of Luminal A, 10.5% (2/19) of luminal B, 71.4% (5/7) of basal-like, and 71.4% (5/7) of HER-2 overexpression samples (Table 1). As expected, cells positive for ER/PR showed a typical nuclear staining pattern (data not shown), whereas HER-2-positive tissues were characterized by a membrane pattern (data not shown). Immunostaining against VASP showed a ubiquitous expression pattern in most epithelial cells and a cytosolic as well as focal pattern in tumors (Figure 1). In addition, patients with basal-like and HER-2-positive subtypes exhibited particularly high VASP expression. Therefore, our data suggests that high expression of VASP correlates with basal-like breast cancer (luminal A or B *vs*. basal-like, P < 0.05) (Table 1).

**Table 1:** Immunohistochemistry analysis of VASP expression in four subtypes of breast cancer

	Cases (n)	VASP			VASP (n%)		
		+	++	+++	+	++	+++
Luminal A	8	3	3	2	37.5	37.5	25.0
Luminal B	19	9	8	2	47.4	42.1	10.5
Basal-like	7	1	1	5	14.3	14.3	71.4
HER-2	7	0	2	5	0	28.6	71.4

n: indicates the sample numbers in that category used for immunohistochemistry staining.+, ++ and +++: indicate weak, moderate, or strong staining intensity, respectively.(n%) represent the percentage of samples with varying degrees positive staining in cytoplasm.

### Berberine inhibits the proliferation of MDA-MB-231 and MCF-7 cells differentially

**Figure 2:**
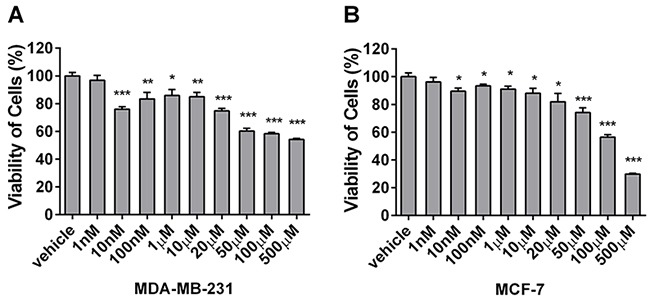
Berberine inhibits the proliferation of MDA-MB-231 and MCF-7 cells MDA-MB-231 and MCF-7 cells were treated with berberine dissolved in DMSO or DMSO as a vehicle control for 24 h. **A.** The viability of MDA-MB-231 cells was determined by MTT assay. **B.** The viability of MCF-7 cells was determined by MTT assay. Each experiment was repeated three times. *P < 0.05, **P < 0.01, ***P < 0.001.

To compare the effects of berberine on the proliferation of basal-like and luminal-type breast cancer cells, MCF-7 (luminal A phenotype) and MDA-MB-231 cells (basal-like phenotype) were treated with different concentrations of berberine, and cell viability was analyzed by MTT assay. MDA-MB-231 cells showed reduced proliferation (76.01% vs. 29.85% without and with treatment, respectively) (Figure 2A). Conversely, MCF-7 cells treated with berberine exhibited a much milder reduction in proliferation (from 93.40% to 56.45%) (Figure 2B). Therefore, our data show that berberine inhibits MDA-MB-231 and MCF-7 cell proliferation, and that this effect is stronger in MDA-MB-231 cells.

### Berberine inhibits the migration of MDA-MB-231 and MCF-7 cells differentially

**Figure 3:**
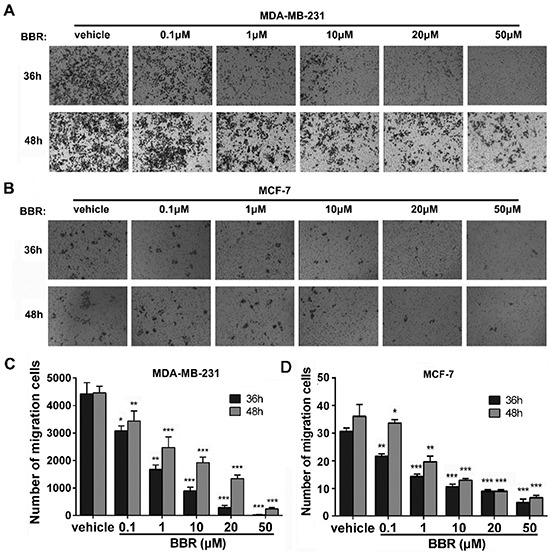
Berberine inhibits the migration of MDA-MB-231 and MCF-7 cells **A.** The migration ability of MDA-MB-231 cells was measured using transwell assay. **B.** The migration ability of MCF-7 cells was measured using transwell assay. **C.** Number of MDA-MB-231 cells migrating through the membrane. **D.** Number of MCF-7 cells migrating through the membrane. Data were expressed as mean ± standard error of the mean from three independent experiments. *P < 0.05, **P < 0.01, ***P < 0.001, *vs.* vehicle control.

To evaluate berberine's inhibitory effect on breast cancer cell migration, we conducted transwell migration assays. Our results show that administration of berberine decreases migration of both MDA-MB-231 and MCF-7 cells in a dosage- and time-dependent manner (Figure 3A and 3B). After 36 h of berberine treatment, the reduction in migration ability of MDA-MB-231 cells ranged between 30% at lower concentration (0.1 μM) and 99% at higher concentration (50 μM). The reduction in migration of MCF-7 cells ranged between 30% at 0.1 μM berberine and 84% at 50 μM berberine (Figure 3C and 3D). Similarly, after 48 h of treatment the reduction in cell migration was between 23% (0.1 μM) and 95% (50 μM) in MDA-MB-231 cells and between 5% (0.1 μM) and 82% (50 μM) in MCF-7 cells (Figure 3C and 3D). Therefore, berberine suppresses migration of MDA-MB-231 cells more efficiently than migration of MCF-7 cells (Figure 3C and 3D). It should be noted that the basal number of invading MCF-7 cells was much lower than that of MDA-MB-231 cells, implying that the latter possess higher migration ability and are therefore more vulnerable to the anti-migration effect of berberine.

### Berberine inhibits basal-like subtype breast cancer growth in a xenograft mouse model

**Figure 4:**
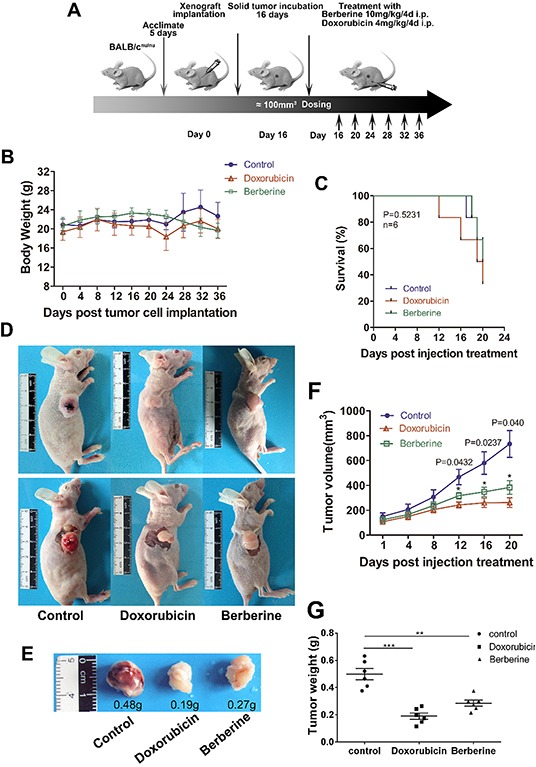
Berberine inhibits tumor growth in the MDA-MB-231 xenograft model **A.** Experimental design of xenograft animal model. **B.** Body weight of xenografted mice. **C.** Kaplan-Meier survival analysis and log-rank test survival of xenografted mice (P > 0.05,). **D.** Representative images of mice with solid tumors. **E.** Representative tumor growth and weight of xenografted mice. **F.** Volumes of tumors from xenografted mice. Data are presented as mean ± standard error of the mean at day 10-36 post-tumor implantation. *P < 0.05. **G.** Weights of solid tumors extracted from sacrificed mice. ***P < 0.001 and **P < 0.01.

To examine the effect of berberine on breast cancer growth *in vivo*, we subcutaneously injected MDA-MB-231 cells into 6 weeks old BALB/c^nu/nu^ athymic mice. When tumor volume reached 80 to 200 mm^3^, the mice were divided into three groups and treated with intraperitoneal injections of placebo, berberine (10 mg/kg) or doxorubicin (4 mg/kg) every four days (Figure 4A). Doxorubicin is the most effective chemotherapy drug for breast cancer treatment; therefore, we used it here as a positive control. Our results show that berberine or doxorubicin did not alter body weight (Figure 4B), and had no effects on the survival of tumor-bearing mice (Figure 4C). However, mice injected with berberine showed decreased tumor volume and reduced tumor weight compared to the control, similarly to doxorubicin (Figure 4D–4G). In particular, the treated tumors displayed a substantial growth delay of 12 days (P < 0.05) compared to controls (Figure 4F). Although doxorubicin showed stronger inhibition of tumor growth than berberine, our results indicate that berberine suppresses the growth of basal-like subtype breast cancer cells *ex vivo*.

### Berberine has no effects on transcription and expression of VASP

**Figure 5:**
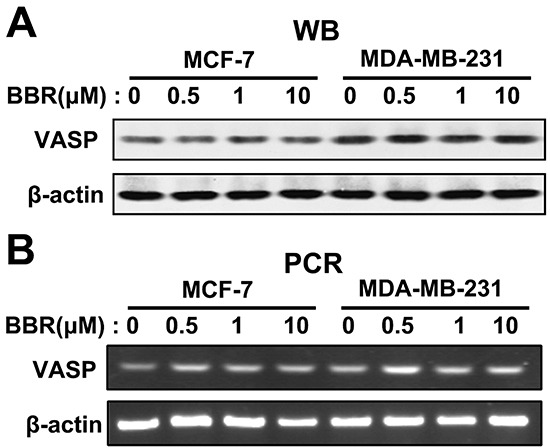
Berberine does not affect expression of VASP MDA-MB-231 and MCF-7 cells were treated with different concentrations of berberine for 48 h. **A.** Western blot analysis of VASP protein level, using β-actin as a loading control. **B.** RT-PCR analysis of VASP mRNA expression level.

Given VASP's role in breast cancer cell motility and berberine's inhibition of cell motility, we tested whether VASP is regulated by berberine. We first measured VASP expression in MDA-MB-231 and MCF-7 cells 48 h after treatment with different concentrations of berberine. Our results show that VASP expression at both the protein (Figure 5A) and the mRNA level (Figure 5B) in either cell line is unaffected by berberine treatment.

### Berberine binds to VASP

**Figure 6:**
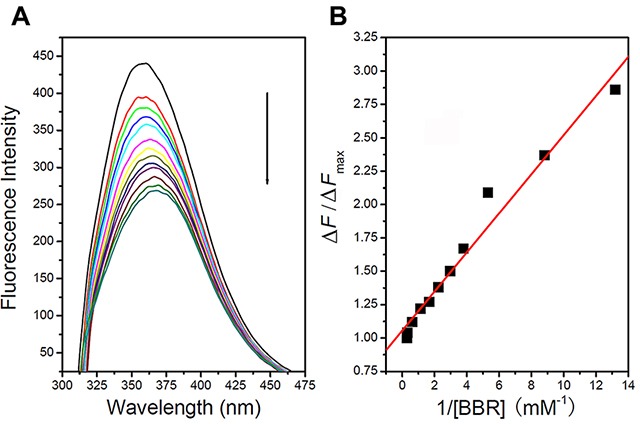
Berberine binds to VASP **A.** When berberine concentration was increased from 0 to 3.4 μM, the fluorescence intensity of VASP decreased and showed no significant shift in the maximum fluorescence emission wavelength. **B.** Plot of fluorescence intensity *vs.* berberine concentration.

To investigate whether berberine binds to the VASP protein, the fluorescence emission spectra of the tryptophan residues in VASP were recorded following excitation at 295 nm. As shown in Figure 6, when the concentration of berberine was increased from 0 to 3.4 μM, the fluorescence intensity of VASP decreased with no significant shift of the maximum fluorescence emission wavelength. The plot of the fluorescence intensity vs. berberine concentration was approximately linear within this range. The *K*d of berberine derived from the binding curve was 1×10^−7^ M, approximately equal to the reciprocal of *K*_b_ (constant binding). These data indicate that berberine directly binds to VASP.

### Berberine targets VASP's EVH1 domain and alters its configuration

**Figure 7:**
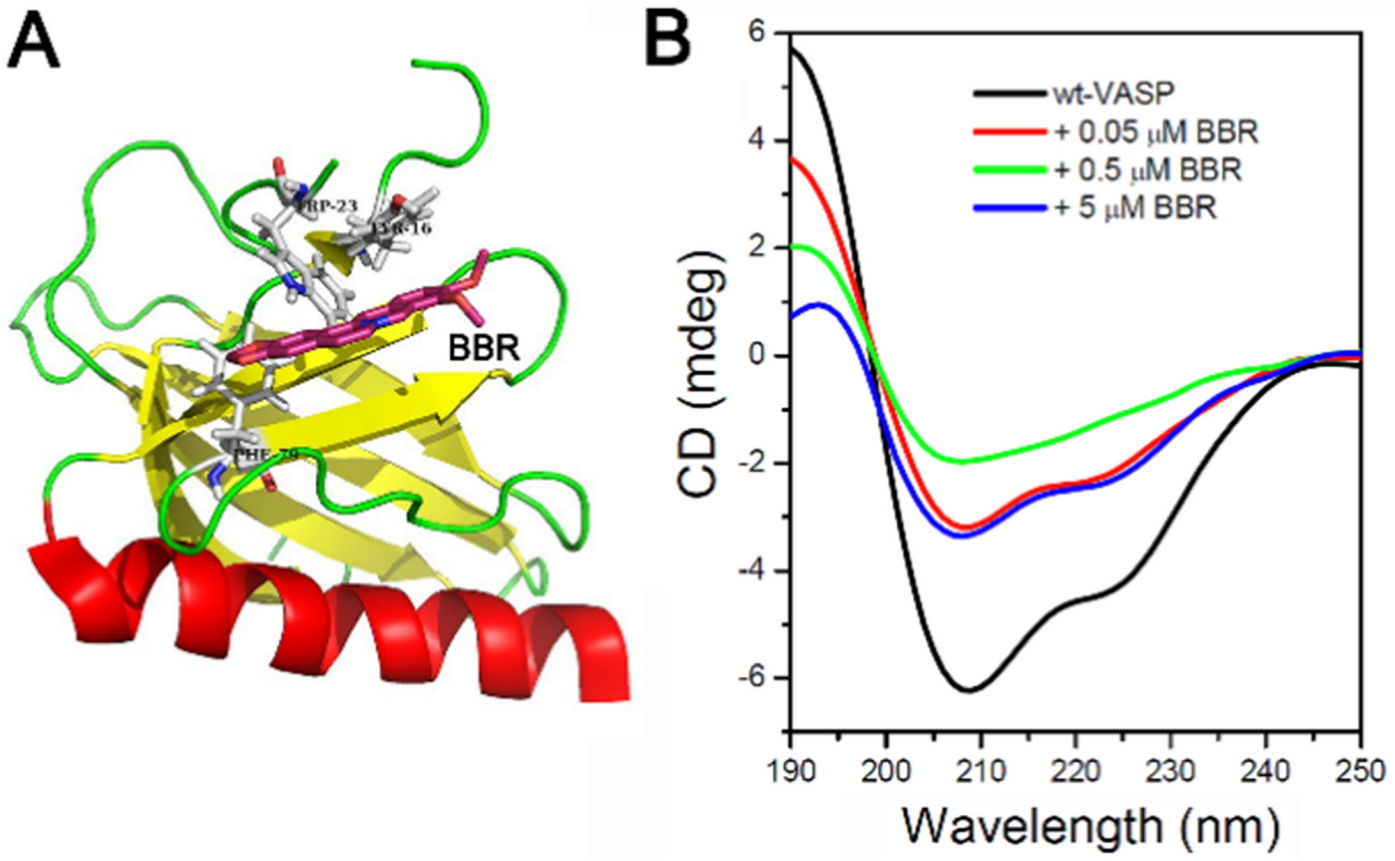
Berberine binds to the EVH1 domain of VASP and affects the far-UV CD spectra of VASP protein **A.** The most favorable binding mode of berberine (berberine) to the EVH1 domain of VASP. **B.** Far-UV CD spectra of VASP protein. The concentration of VASP was 2.18 μM. Berberine concentration ranged from 0 to 5 μM. Band width was 1 nm, response was 2 s, measurement range was 250-190 nm, data pitch was 1 nm, scanning speed was 200 nm/min, and cell length was 0.1 cm.

We next aimed to determine the exact binding site of berberine on the VASP protein. We employed the AutoDock software to configure the most favorable binding site of berberine on the EVH1 domain of VASP, which contains a hydrophobic cluster. The most favorable binding mode of berberine (purple structure) on the EVH1 domain of VASP is shown in Figure 7A. Furthermore, the far-UV CD spectrum of VASP alone exhibited an intense negative maximum at 208 nm and a less intense negative maximum at 222 nm, suggesting the presence of a significant amount of α-helical structure. However, in the presence of berberine (0.05 μM), the negative maxima at both 208 and 222 nm were reduced, suggesting a decrease of α-helical structure. When the concentration of berberine reached 0.5 μM, the negative maximum at 222 nm was no longer detectable. However, a partial recovery of the negative maximum at 222 nm was observed when the concentration of berberine was increased to 5 μM (Figure 7B). Collectively, these results indicate that berberine binds to VASP's EVH1 domain and alters its conformation.

### Berberine inhibits actin polymerization via VASP

**Figure 8:**
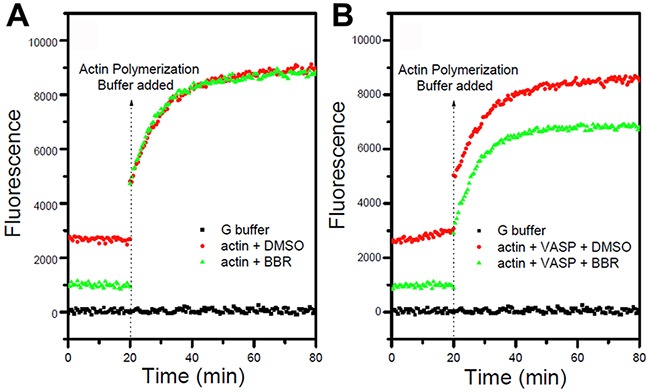
Berberine inhibits actin polymerization in the presence of VASP Polymerization was determined according to increasing pyrene fluorescence. Berberine (berberine) had a quenching effect on the fluorescence signal of pyrene-actin. **A. Berberine** had no effect on actin polymerization with the addition of actin polymerization buffer. **B.** In the presence of 0.5 μM VASP, the inhibitory effect of berberine remained and reduced the final amount of F-actin.

**Figure 9:**
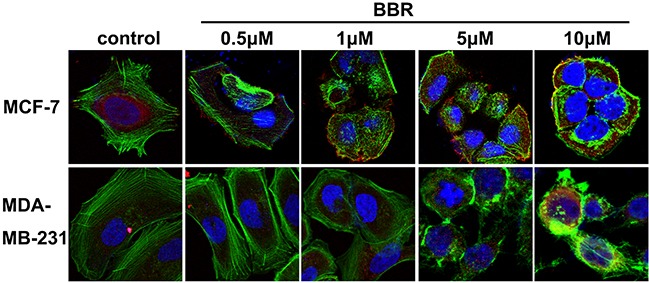
Berberine inhibits the formation of F-actin Representative confocal microscopy images from 3 separate experiments of MDA-MB-231 and MCF-7 cells 3 h after berberine treatment (0.5-10 μM). F-actin (stained with phalloidin): green; VASP: red; and nuclei: blue. Original magnification ×1,000.

Having demonstrated that berberine binds to the VASP, we next tested whether this interaction interfered with VASP's activity. F-actin examined by pyrene fluorescence was used as an indicator of actin assembly. F-actin exhibited dynamic instability (auto assembly-disassembly) in the absence of VASP (no purified VASP added to the reaction system) but polymerized more stably upon VASP addition in MDA-MB-231 and MCF-7 cells (Figure 8A). As expected, berberine reduced the amount of F-actin and destabilized F-actin filaments in a dosage-dependent manner, indicating an inhibitory effect of berberine on actin assembly in these breast cancer cells (Figure 8B). Consistently, fewer actin aggregates and bundled stress fibers were observed with increasing berberine concentration up to 1 M, whereas stress fibers gathered by F-actin appeared long and regular in control cells (Figure 9). Moreover, no stress fibers were observed in the cortical pool with only some irregular actin filaments scattered around the cell periphery even when the berberine concentration was as low as 5 μM (Figure 9). Also, the intensity and distribution of cytoplasmic VASP was not altered (Figure 9). Taken together, these data suggest that berberine inhibits actin polymerization by blocking VASP activity in breast cancer cells.

## DISCUSSION

Breast cancer is one of the malignancies with highest incidence and mortality, causing ~411,000 yearly deaths among women worldwide [21, 22]. Basal-like breast cancer is resistant to endocrine and targeted Herceptin therapies, making it one of the most difficult types of cancer to treat, with poor prognosis [10] even after routine systemic chemotherapy treatment [9].

Although previous studies have revealed many potential biomarkers associated with poor prognosis in basal-like breast cancer, including basal cytokeratins (CK5/6, CK14, CK17), epidermal growth factor receptor (EGFR), c-kit, P63, P-cadherin and FOXC1, currently, there are no targeted treatments. Elevated levels of the actin-associated protein VASP in lung adenocarcinoma correlate with the degree of de-differentiation and poor pathological stage [23]. Moreover, Liu *et al.* showed that VASP overexpression promotes neoplastic transformation in wild-type NIH 3T3 cells [24]. Furthermore, we previously showed that VASP promotes breast cancer cell migration and that VASP levels are higher in basal-like than in luminal breast cancer [17, 18]. In addition, VASP levels are higher in basal-like MDA-MB-231 cells than in luminal type MCF-7 cells. These data suggest that VASP may serve as a therapeutic target to treat basal-like breast cancer.

VASP is a widespread actin-related protein that locates to highly dynamic regions of the cell membrane, and participates in biological processes that depend on actin (F-actin) assembly/disassembly, such as adhesion, spreading, migration, invasion and metastasis [25–28]. The VASP protein consists of three functional domains: an Ena/VASP homology 1 (enabled/vasodilator-stimulated phosphoprotein homology 1, EVH1) domain at the N-terminus, a central proline-rich region (PRR), and an Ena/VASP homology domain 2 (enabled/vasodilator-stimulated phosphoprotein homology 2, EVH2) at the C-terminus [29]. The N-terminal EVH1 domain mediates VASP anchoring to promote actin filament assembly by recognizing “FPPPP” motifs within other cytoskeleton-associated proteins [30]. This allows for the recruitment of VASP by various proteins to cell-cell junctions [31], cell-extracellular matrix adhesion plaques [32], the end of actin filaments [29], and bacterial membrane surfaces [33]. The VASP EVH1 domain possesses a hydrophobic β-barrel fold, with seven β-strands packed together to form an antiparallel β-sandwich at the N terminus, and an α-helix at the C-terminus. EVH1-mediated recognition and anchoring is a prerequisite for recruitment of profilin-actin complexes by the PRR region [26, 34] and binding of actin filaments by the EVH2 domain [27, 29]. These interactions, enable VASP to promote actin filament elongation [29, 35] and cell migration. On the other hand, berberine inhibits cell migration. Therefore, we hypothesized that berberine acts as a tumor suppressor by binding VASP to impair cell migration in breast cancer cells.

Berberine is a derivative of quaternary ammonium with conjugated double bonds. Its structure is characterized by the combination of two isoquinolines. Our structural and electronic distribution analyses show unpaired electron orbits in the oxygen atoms of the 1,2-methylenedioxy, vertically aligned with the electron orbit of benzene ring A and a positive charge of the N atom, which confers slight hydrophilicity. Therefore, we hypothesized that berberine specifically binds to the gap in VASP's EVH1 region, thereby inhibiting VASP's ability to promote actin assembly. Indeed, we found in our present study that although berberine does not affect VASP protein and mRNA levels (Figure 4), it can bind VASP and affect its structure (Figure 6 and 7), thereby inhibiting actin polymerization (Figure 8 and 9).

Previous studies showed that 100-500 μM berberine (pretreatment for 24 h) inhibits the growth of breast cancer cells by more than 50% [25, 36]. Additionally, 10 μM berberine inhibits migration in breast cancer MCF-7 and MDA-MB-231 cells [25, 37, 38]. Our current study confirms such findings. In addition, our data show that basal-like breast cancer MDA-MB-231 cells are more sensitive to berberine's inhibitory effects on cell migration than MCF-7 cells. Furthermore, we propose a molecular mechanism for this inhibition, showing that berberine inhibits VASP and thereby actin polymerization. Importantly, the concentrations of berberine used in our studies (0.1-50 μM) are physiologically relevant since the concentration of berberine in human plasma remains low (~40 μM) at conventional oral dosage [36].

Various mechanisms have been proposed for berberine's tumor suppressor functions in several types of cancer, including colonic adenomas and adenocarcinomas [39, 40], esophageal cancer [41], colon cancer [42], nasopharyngeal carcinoma [43], and lung cancer [36]. Although these studies revealed that berberine can exert antitumor effects through many different pathways, including the promotion of senescence [44–46] and apoptosis [14] in cancer cells, as well as tumor microenvironment remodeling [47], further studies are needed to propose a comprehensive model that explains the antitumor actions of berberine in various types of cancer.

Here, we elucidated a novel mechanism by which berberine inhibits basal-like breast cancer cell proliferation and migration. Our findings provide a rationale for clinical trials to treat basal-like breast cancer, a very aggressive and treatment-resistant type of cancer.

## MATERIALS AND METHODS

### Cell culture

The breast cancer cell lines MDA-MB-231 and MCF-7 were obtained from Cell Bank of Type Culture Collection of Chinese Academy of Sciences (Shanghai, China). MDA-MB-231 cells were cultured in RPMI-1640 medium (HyClone, Logan, UT, USA) supplemented with 10% fetal bovine serum (FBS) (HyClone, Logan, UT, USA), 100 U/mL penicillin and 100 μg/mL streptomycin under the conditions of 37°C and 5% CO_2_. MCF-7 cells were maintained in Eagle's minimum essential medium (MEM) supplemented with 10% FBS, 100 U/mL penicillin and 100 μg/mL streptomycin.

### MTT assay

Cell viability was determined using MTT assay (Amresco, Solon, OH, USA). Briefly, 5×10^4^/ml cells per well were plated in 96-well plates and incubated for 24 h. The cells were then treated with different concentrations (0, 1 nM, 10 nM, 100 nM, 1 μM, 10 μM, 20 μM, 50 μM, 100 μM and 500 μM) of berberine (Sigma-Aldrich, St. Louis, MO, USA) for 24 h. Then the cells were treated with 20 μl of 5 mg/ml MTT and incubated for 4 h at 37°C. Then the medium was discarded, and 200 μl of dimethylsulfoxide (DMSO) (Sigma-Aldrich, St. Louis, MO, USA) was added. Absorbance was measured at 570 nm with an ELISA plate reader (Infinite® 200 PRO, TECAN, Männedorf, CH). The viability of berberine-treated cells was calculated by comparing to vehicle-treated cells, which were arbitrarily assigned 100%. The experiments were performed in triplicate, independently.

### Cell migration assay

Migration of MDA-MB-231 and MCF-7 cells was evaluated using 6.5 mm Transwell® chambers with 8.0 μm pores size polycarbonate membrane filters (Corning Costar, Corning, NY). Cells (2.5 ×10^4^) suspended in 0.1 ml serum-free medium in the presence or absence of berberine were seeded onto the upper chamber. Half a milliliter of medium containing 10% FBS was added to the bottom chamber. After 36 h or 48 h treatment with berberine, filter inserts were removed from the wells. The cells on the upper surface of the filter were wiped with a cotton swab. Filter membranes were stained with 0.1% crystal violet (Sigma-Aldrich, St. Louis, MO, USA) for 30 min, and the invaded cells were quantified by counting 5 fields at a magnification of ×100. Each experiment was repeated in triplicate and the mean value was computed.

### Clinical samples

This study was approved by Ethics Commission of the School of Medicine of Wuhan University. Breast cancer samples (n=41) were obtained from patients recruited at Zhongnan Hospital of Wuhan University between January 1, 2009 and December 30, 2012, who had never been treated with anti-cancer drugs or neoadjuvant chemotherapy. The samples were sectioned and fixed overnight in 10% buffered formalin. The samples were immunohistochemically stained for ER, PR, and HER-2 and were independently interpreted by 2 pathologists. Invasive breast carcinomas with weak, moderate, or strong nuclear labeling for ER or PR in greater than 1% of cells were considered ER+ or PR+, respectively, in accordance with the ASCO/CAP ER and PgR guideline [48]. Her2+ samples had a 3+ (strong positive) immunohistochemical score or a Her2 fluorescence *in situ* hybridization amplification ratio > 4 [49]. Samples with ambiguous ratios (1.8–2.2) or low-level amplification (ratios, 2.2–4.0) were excluded.

### Immunohistochemical analysis

For paraffin-embedded breast cancer tissues, 5 μm-thick sections were deparaffinized in xylene, followed by treatment with a graded series of alcohol (100, 95, and 80%, v/v) and rehydration in PBS (pH 7.5). Sections were microwaved 5 min for antigen retrieval and incubated with 3% hydrogen peroxide in methanol (v/v) for 12 min to block endogenous peroxidase. Next, the sections were washed with PBS (pH 7.5), and incubated in protein blocking solution (0.5% normal goat serum in PBS, v/v) for 30 min. The sections were then incubated with antibody against human VASP (1:200, ENZO Life Sciences, Farmingdale, NY) in a humidified chamber for 2 h at 37°C, rinsed with PBS 3 times, and incubated with peroxidase-conjugated secondary antibody (1:200, Jackson ImmunoResearch, West Grove, PA) for 1 h at room temperature. To detect positive reactions, the slides were incubated with stable 3,39-diaminobenzidine (DAB) for 5-10 min (Zhongshan Jingqiao Biotechnology, Beijing, China). The sections were rinsed with distilled water, counterstained with Gill's hematoxylin for 1 min (Sigma, St. Louis, MO), and observed under a BX53 Olympus microscope. Mean density of the sections was analyzed using Image-Pro Plus 4.5 software. The staining intensity of VASP was scored as negative (−), weak (+), moderate, (++), or strong (+++), as described previously [7, 50].

### Western blot analysis

Cells were harvested at the indicated time and lysed using routine procedures. Proteins were separated on a 10% SDS-polyacrylamide gel and transferred to polyvinylidene difluoride membranes. The membranes were then blotted with antibody for VASP (1:1000, ENZO Life Sciences, Farmingdale, NY, USA) or β-actin (1:500, Santa Cruz Biotechnology, Santa Cruz, CA, USA). The signal was visualized with an alkaline phosphatase-conjugated anti-rabbit or mouse IgG antibody (1:2000, Jackson ImmunoResearch, West Grove, PA). All experiments were performed in triplicate.

### Reverse transcription PCR (RT-PCR)

Total RNA was extracted from cells using Trizol reagent (Invitrogen, Carlsbad, CA). Total RNA (3 μg) was used for first-strand cDNA synthesis with RevertAidTM First Strand cDNA Synthesis Kit (Fermentas, Vilnius, LTU). PCR amplification of cDNA was performed using the following specific primers: VASP: forward: 5′-GCGCCTGGTACCATGACGCAAGTTGGGGAGAAAACC-3′ and reverse:

5′-GCG CCTGGATCCCAGGGAGAACCCCGCTTCC-3′; β-actin forward:

5′-CATTAAGGAGAAGCTGTGCT-3′ and reverse: 5′-GTTGAAGGTAGTTT CGTGGA -3′. The PCR products were visualized by electrophoresis on a 2% agarose gel (w/v).

### Intrinsic fluorescence spectroscopy

VASP protein was expressed in *E. coli Rosetta* by induction with 0.5 mM isopropyl-1-thio-β-D-galactopyranoside (IPTG) at 18°C for 16 h as previously described [51, 52]. The intrinsic fluorescence spectroscopy was used to investigate the interaction between berberine with VASP at 25°C by LS-55 luminescence spectrometer (Perkin-Elmer Life Sciences, Shelton, CT). An excitation wavelength of 295 nm was used for intrinsic fluorescence measurements, and the emission spectra were recorded between 300 and 475 nm. The excitation and emission slits were both at 15 nm, and the scan speed was 1,000 nm/min. Six hundred μl of VASP (0.4 μM) were placed in a 1 mm thermostated quartz fluorescence cuvette and successively titrated with 22.8 μM, 91.2 μM and 0.18 mM berberine with continuous stirring. The fluorescence measurements were performed with protein samples that had an optical density at 280 nm of less than 0.1 to avoid the inner filter effects [53]. Buffer titrated with an equivalent amount of berberine was also measured under the same conditions as control to analyze fluorescence of the samples. Each spectrum was measured three times to acquire the final fluorescence emission spectra. The plot was fitted to the following equation: Δ*F*/Δ*F*max = [berberine]/([berberine] + *K*d), where Δ*F* is the change of fluorescence intensity after each injection, Δ*F*max is the maximum fluorescence intensity change, and [berberine] is the concentration of berberine.

### Docking of berberine to EVH1 domain of VASP

The data for the computational modeling in this study were derived from the recent NMR structure of EVH1 (PDB ID 1EGX). The best representative conformer (structure 1) in this ensemble (total of 20 structures) was chosen for docking and assignment with Kollman-UTI charges in the Amber 4.0 force field. The structure of BER was manually constructed according to the standard bond lengths and angles, minimized by a molecular mechanical method and then optimized in the Tripos force field with Gasteiger-Hückel charges. The docking calculations were performed with the software package AutoDock Vina [54], in which the Lamarckian Genetic Algorithm was applied. In each calculation, the initial structure of the ligand was in an arbitrary conformation as well as orientation and position. After 100 runs, the most favorable binding model was selected according to the binding energy and geometry match.

### Circular dichroism (CD) spectroscopy

The far-UV CD spectra were measured with a Jasco J-810 spectropolarimeter (Jasco Corporation, Tokyo, Japan) using a 0.1 cm path length cylindrical cell at 190-250 nm (protein secondary structure) at 25°C. The band width was set to 1 nm, while the response time was 1 s. VASP was thoroughly mixed in buffer (50 mM Tris-HCl, pH 7.5, 100 mM NaCl, 1 mM tris-(2-carboxyethyl) phosphine, TCEP) with different concentrations of berberine and allowed to equilibrate thermally for 4 min prior to the CD measurements. Each sample spectrum was corrected by subtracting from the spectrum (baseline) recorded for the buffer containing an equivalent concentration of berberine. The relative change in the α-helical content of VASP, represented by the relative change in molar ellipticity of VASP at 222 nm, was calculated from the CD spectra [55]. Each spectrum was an average of three different scans obtained by collecting data at 1 nm intervals with a scan speed of 200 nm/min.

### Actin polymerization assay

Actin polymerization assay was performed using the kit from Cytoskeleton (Denver, CO, USA). Briefly, pyrene actin (0.4 mg/ml, 200 μl) in G-buffer (5 mM Tris, 0.2 mM CaCl2, and 0.2 mM ATP, pH 8.0) was prepared according to the manufacturer's recommendations. The actin polymerization reaction system was mixed with 20 μl of 5 μM VASP in 1× KMEI (50 mM KCl, 1 mM MgCl_2_, 1 mM EGTA, and 10 mM imidazole, pH 7.0) in the presence or absence of 5 μM berberine was added to (The final concentration of VASP and berberine were 0.5 μM). After 20 min, polymerization reaction was initiated by adding 20 μl 10 × actin polymerization buffer (0.5 mM KCl, 20 mM MgCl_2_, 10 mM ATP) to convert Ca^2+^-ATP-actin to Mg^2+^-ATP-actin. Pyrene fluorescence was measured with a Spectramax M2 Microplate Reader (Molecular Device) with an excitation wavelength of 360 nm and an emission wavelength of 410 nm.

### Immunofluorescence staining

MCF-7 and MDA-MB-231 cells were cultured on coverslips in a 24-well plate. Cells were fixed with 4% paraformaldehyde for 30 min at room temperature, washed, and permeabilized with 0.5% Triton X-100 for 5 min. The cells were incubated with antibody for VASP (1:100, ENZO Life Sciences, Farmingdale, NY, USA) at 4°C overnight followed by incubation with secondary antibody conjugated to TRITC (1:100, Jackson ImmunoResearch, West Grove, PA, USA). Double staining was conducted continuously with phalloidin-Alexa 488 (1:40, Invitrogen, Carlsbad, CA). ProLong Gold Antifade reagent along with DAPI (Invitrogen) was used to mount the coverslips to slides.

### *In vivo* tumor xenograft model

Animal experiments were performed following the Guide for the Care and Use of Laboratory Animals of Wuhan University. Female athymic mice 6 weeks of age (nu/nu) on a BALB/c background (Hunan SJA Laboratory, Permission number: HNASLKJ20120623) were raised in a pathogen-free isolation facility with a light/dark cycle of 12/12 h and *ad libitum* fed with rodent chow and water. MDA-MB-231 cells (1×10^7^) in 200 μl culture medium were subcutaneously injected into the right hind flank. Animal-bearing tumors were randomly assigned to treatment groups (6 mice per group). When tumor grew to 100 mm^3^, the mice were divided into 3 subgroups. The first group received an intraperitoneal injection of berberine (10 mg/kg) every 4 days, while the second group received an injection of vehicle (DMSO) only, and the third group received injection of doxorubicin (4 mg/kg). Mice were monitored up to 12-20 days after initiation of treatment. Tumor size was monitored by measuring the length and width with calipers every 4 days, and volumes were calculated with the formula: L×W^2^/2 = mm^3^, where L is the length and W the width of the tumor [10]. Kaplan-Meier survival analysis and log-rank test were used to analyze mice survival. When the mice were sacrificed, the tumors and livers were fixed in 4% paraformaldehyde, sectioned, and stained with hematoxilin-eosine for light microscopic analysis.

### Statistical analysis

All results were presented as the mean ± standard error of mean. Statistical analysis of data having equal variance was performed by one-way or two-way analysis of variance (ANOVA) followed by Tukey's Post Hoc test when appropriate. Chi-square test was used for intergroup comparison for immunohistochemical analysis of VASP expression, considering *p* < 0.05 as statistically significant.
